# Use of the GSK MenACWY-CRM/MenB-4C Pentavalent Meningococcal Vaccine Among Persons Aged ≥10 Years: Recommendations of the Advisory Committee on Immunization Practices — United States, 2025

**DOI:** 10.15585/mmwr.mm7501a2

**Published:** 2026-01-08

**Authors:** Avnika B. Amin, Jennifer P. Collins, Xiaoyu Dong, Andrew J. Leidner, Jamie Loehr, Charlotte A. Moser, Lucy A. McNamara

**Affiliations:** ^1^Division of Bacterial Diseases, National Center for Immunization and Respiratory Diseases, CDC; ^2^Epidemic Intelligence Service, CDC; ^3^Immunization Services Division, National Center for Immunization and Respiratory Diseases, CDC; ^4^Cayuga Family Medicine, Ithaca, New York; ^5^Children’s Hospital of Philadelphia, Philadelphia, Pennsylvania.

**Summary**

**What is already known about this topic?**

Meningococcal disease is a serious bacterial infection caused by *Neisseria meningitidis*. A new pentavalent meningococcal vaccine (MenACWY-CRM/MenB-4C [Penmenvy, GSK]) protects against *N. meningitidis* serogroups A, B, C, W, and Y and is licensed for use in persons aged 10–25 years. MenACWY-CRM/MenB-4C is the second pentavalent meningococcal vaccine approved in the United States.

**What is added by this report?**

On April 16, 2025, the Advisory Committee on Immunization Practices recommended that, when both quadrivalent (serogroups A, C, W, and Y) and serogroup B meningococcal (MenB) vaccine are indicated concurrently for persons aged ≥10 years, MenACWY-CRM/MenB-4C may be administered instead.

**What are the implications for public health practice?**

Because different manufacturers’ serogroup B–targeting vaccines are not interchangeable, this recommendation provides a pentavalent vaccine option for persons receiving the GSK MenB vaccine (MenB-4C) for other doses.

## Abstract

Meningococcal disease is a serious bacterial infection caused by *Neisseria meningitidis*. Serogroups B, C, W, and Y cause the majority of cases of this disease in the United States. These serogroups are targeted by different meningococcal vaccines available in the United States. Two quadrivalent (serogroups A, C, W, and Y) meningococcal conjugate vaccines (MenACWY) (MenACWY-CRM [Menveo, GSK] and MenACWY-TT [MenQuadfi, Sanofi Pasteur]) and two serogroup B meningococcal vaccines (MenB) (MenB-4C [Bexsero, GSK] and MenB-FHbp [Trumenba, Pfizer]) are licensed for use in the United States and recommended by CDC’s Advisory Committee on Immunization Practices (ACIP). Indications for MenACWY and MenB vaccination have not changed since indications for their use were published in 2020. A pentavalent (serogroups A, B, C, W, and Y) meningococcal vaccine (MenABCWY) (MenACWY-TT/MenB-FHbp [Penbraya, Pfizer]) has been licensed and recommended for use since October 2023. On February 14, 2025, the Food and Drug Administration licensed a second pentavalent MenABCWY vaccine (MenACWY-CRM/MenB-4C [Penmenvy, GSK]) for prevention of invasive disease caused by *N. meningitidis* serogroups A, B, C, W, and Y in persons aged 10–25 years, the same indication for which MenACWY-TT/MenB-FHbp is licensed. On April 16, 2025, ACIP recommended that MenACWY-CRM/MenB-4C may be used when both MenACWY and MenB are indicated at the same visit for 1) healthy persons aged 16–23 years (routine schedule) when shared clinical decision-making favors administration of MenB vaccine and 2) persons aged ≥10 years who are at increased risk for meningococcal disease (e.g., because of persistent complement deficiencies, complement inhibitor use, or functional or anatomic asplenia). Different manufacturers’ serogroup B–targeting vaccines are not interchangeable; therefore, when MenACWY-CRM/MenB-4C is used, MenB-4C should be used for the other MenB doses. This report summarizes evidence considered for these recommendations and provides clinical guidance for the use of MenACWY-CRM/MenB-4C.

## Introduction

Meningococcal disease, caused by *Neisseria meningitidis*, is a serious bacterial infection that can cause invasive or noninvasive disease. Serogroups B, C, W, and Y cause the majority of cases of meningococcal disease in the United States (*1*). Risk factors for meningococcal disease include anatomic or functional asplenia, persistent complement component deficiencies, use of complement inhibitors (e.g., eculizumab or ravulizumab), HIV infection, active or passive exposure to tobacco smoke, and recent upper respiratory tract infection (*1*). Persons living in crowded settings, such as college residence halls, and those who are close contacts of persons with meningococcal disease are also at increased risk for acquiring meningococcal disease (*1*).

CDC’s Advisory Committee on Immunization Practices (ACIP) recommends administration of a single dose of quadrivalent (serogroups A, C, W, and Y) meningococcal vaccine (MenACWY) to persons aged 11–12 years, with a booster dose at age 16 years, as part of the routine childhood immunization schedule. For persons aged ≥2 months who are at increased risk for meningococcal disease because of certain medical conditions or other exposures, ACIP recommends a multiple-dose MenACWY series, with regular booster doses if the recipient remains at increased risk (Box) (*2*). Two MenACWY vaccines (MenACWY-CRM [Menveo, GSK] and MenACWY-TT [MenQuadfi, Sanofi Pasteur]) are licensed and recommended for use in the United States. In addition, ACIP recommends serogroup B meningococcal vaccine (MenB) as a 2-dose series for healthy persons aged 16–23 years based on shared clinical decision-making (e.g., given the estimated relatively short [1–2 years] duration of MenB protection and the high cost per quality-adjusted life year [QALY] gained), and for risk-based administration to persons aged ≥10 years (*2*). Two MenB vaccines (MenB-4C [Bexsero, GSK] and MenB-FHbp [Trumenba, Pfizer]) are licensed and recommended for use in the United States.

BOX **Meningococcal vaccination recommendations — Advisory Committee on Immunization Practices, United States, 2025**

The Advisory Committee on Immunization Practices (ACIP) recommends quadrivalent (serogroups A, C, W, Y) meningococcal conjugate (MenACWY) vaccination* **for the following groups:**

- **Healthy persons aged 11–12 years**: Routine vaccination with a single dose for all persons aged 11–12 years, with a booster dose at age 16 years
- **Persons aged ≥2 months who are at increased risk for meningococcal disease**: Routine and booster vaccination. (Dosing schedule varies by age and indication; interval for booster doses varies by age.)^†^
  - Persons with certain medical conditions, including anatomic or functional asplenia, HIV infection, and persistent complement component deficiency, and those who use complement inhibitors (e.g., eculizumab or ravulizumab)
  - Persons traveling to countries with hyperendemic or epidemic meningococcal disease, including countries in the African meningitis belt or during Hajj
  - First-year college students who live in residential housing, if not previously vaccinated
  - Military recruits
  - Microbiologists routinely exposed to *Neisseria meningitidis* isolates
  - Persons at increased risk during an outbreak of meningococcal disease caused by serogroup A, C, W, or Y

**ACIP recommends serogroup B meningococcal (MenB) vaccination****^§^** **for the following groups:**

- **Healthy persons aged 16–23 years**: Vaccination with a 2-dose MenB series on the basis of shared clinical decision-making, with 16–18 years the preferred age for MenB vaccination
- **Persons aged ≥10 years who are at increased risk for meningococcal disease**: Routine and booster vaccination. (Dosing schedule varies by indication; the first booster dose should be given 1 year after the primary series, with additional boosters every 2–3 years if the risk remains.)
  - Persons with certain medical conditions, including anatomic or functional asplenia and persistent complement component deficiency, and those who use complement inhibitors (e.g., eculizumab or ravulizumab)
  - Microbiologists routinely exposed to *N. meningitidis* isolates
  - Persons at increased risk during an outbreak of meningococcal disease caused by serogroup B

**ACIP recommends pentavalent (serogroups A, B, C, W, and Y) meningococcal (MenABCWY) vaccination**^¶^ **for the following groups:**

- **Persons aged ≥10 years for whom MenACWY and MenB are both indicated:** Primary series vaccination if MenACWY and MenB would be administered as separate vaccines at the same health care visit
- **Persons aged ≥10 years who are at increased risk for meningococcal disease who previously received MenABCWY or MenB ≥6 months earlier**: Booster vaccination if MenACWY and MenB would be administered as separate vaccines at the same health care visit; if the previous doses included a MenB-FHbp component, MenACWY-TT/MenB-FHbp would be used, whereas MenACWY-CRM/MenB-4C would be used if the previous doses included a MenB-4C component

* Quadrivalent MenACWY vaccines from different manufacturers are interchangeable, although the same vaccine product is recommended for all doses.

^†^ Meningococcal Vaccination | Recommendations of the Advisory Committee on Immunization Practices, United States, 2020

^§^ MenB vaccines from different manufacturers are not interchangeable. All doses used for the primary series (and booster doses, if applicable) should be from the same manufacturer.

^¶^ Pentavalent MenABCWY vaccines from different manufacturers are not interchangeable because the MenB components are not interchangeable. If a serogroup B–targeting vaccine has already been administered, subsequent doses should be from the same manufacturer.

Since October 25, 2023, ACIP has recommended that a pentavalent (serogroups A, B, C, W, and Y) meningococcal vaccine (MenABCWY) (MenACWY-TT/MenB-FHbp [Penbraya, Pfizer]) may be used when both MenACWY and MenB are indicated at the same visit for 1) healthy persons aged 16–23 years when shared clinical decision-making favors MenB administration and 2) persons aged ≥10 years who are at increased risk for meningococcal disease (*3*). Different manufacturers’ serogroup B–targeting vaccines are not interchangeable; therefore, persons who receive pentavalent MenACWY-TT/MenB-FHbp should have already received or should next receive MenB-FHbp for other MenB doses.

On February 14, 2025, a second pentavalent meningococcal vaccine (MenACWY-CRM/MenB-4C [Penmenvy, GSK]) was licensed for use in persons aged 10–25 years (*4*). Both pentavalent vaccines are licensed for the same indications. MenACWY-CRM/MenB-4C contains the same components as those in two existing meningococcal vaccines licensed for use in the United States: 1) *N. meningitidis* serogroup A, C, W, and Y capsular polysaccharides conjugated to CRM197 (MenACWY-CRM [Menveo, GSK]) and 2) *N. meningitidis* serogroup B outer-membrane recombinant proteins (factor H binding protein [fHbp], *Neisseria* adhesin A [NadA], and neisserial heparin binding antigen [NHBA]) and an outer-membrane vesicle component containing porin A (PorA) (MenB-4C [Bexsero, GSK]). This report summarizes the evidence considered for MenACWY-CRM/MenB-4C vaccine recommendations and provides clinical guidance for use.

## Methods

### Meningococcal Vaccines Work Group Activities

During January 2024–March 2025, the ACIP Meningococcal Vaccines Work Group held monthly or bimonthly conference calls to review meningococcal disease epidemiology and evidence regarding the use of MenACWY-CRM/MenB-4C in persons who are currently recommended to receive both MenACWY and MenB (policy question 1), MenACWY only (policy question 2), or MenB only (policy question 3). These policy questions were chosen for parity with a previous evaluation of MenACWY-TT/MenB-FHbp (*3*).

### Review of Meningococcal Disease Epidemiology and Evidence Regarding Vaccine Use

To guide deliberations, ACIP used the Evidence to Recommendations framework and considered the importance of invasive meningococcal disease as a public health problem, benefits and harms of MenACWY-CRM/MenB-4C, values of the target population, acceptability, resource use, health equity, and feasibility (ACIP Evidence to Recommendations for Use of GSK’s Pentavalent Meningococcal Vaccine | MenACWY-CRM/MenB-4C). ACIP evaluated the available evidence on prespecified benefits and harms, each with a ranked importance, using the Grading of Recommendations, Assessment, Development, and Evaluation (GRADE) approach (GSK’s Pentavalent Meningococcal Vaccine | MenACWY-CRM/MenB-4C | GRADE). Critical outcomes included disease caused by serogroups A, B, C, W, and Y; short-term immunity; and serious adverse events. Important outcomes included persistence of immunity, interference with other recommended vaccines administered concurrently, and nonserious adverse events. Evidence for these outcomes was identified through a systematic review (*5*). During the evidence review, the recommended interval between MenB-4C doses for healthy persons aged 16–23 years was increased from ≥1 month to ≥6 months based on new data suggesting better immunogenicity with a longer dosing interval (*6*). This change raised the immunogenicity standard used for policy questions 1 and 3, as information from study arms that received MenB-4C on the new dosing interval was considered.

## Summary of Evidence for Use of  MenACWY-CRM/MenB-4C in Persons Aged ≥10 Years

### Studies Included in Safety and Immunogenicity Assessment

The evidence comprised data from seven multisite randomized controlled trials assessing immunogenicity and safety among healthy participants aged 10–25 years. Evidence for two outcomes (disease caused by serogroups A, B, C, W, and Y and interference with other recommended vaccines administered concurrently) was lacking.

Trials were conducted in Argentina, Australia, Canada, Chile, Colombia, Czechia, Estonia, Finland, Panama, Poland, Turkey, and the United States (*7*–*13*). Depending on the trial, participants who had or had not previously received MenACWY vaccine were included. All study participants had never received MenB vaccine. Across the different trials, participants were randomized to either a pentavalent vaccine group (≥2 doses of MenACWY-CRM/MenB-4C, with or without a booster dose in longer-term extension studies among participants in original studies) or to an active control group reflecting real-world meningococcal vaccination schedules. The control group or groups in each trial varied (Figure). For the safety and immunogenicity assessment, control group participants were classified into one of three groups: 1) 1 dose of only MenACWY-CRM (*7*–*9*,*11*–*13*); 2) ≥2 doses of only MenB-4C, with or without a booster dose in extension studies (*8*,*10*,*11*,*13*); or 3) 2 doses of concomitantly administered MenACWY-CRM + MenB-4C (*5*,*11*).

**FIGURE:**
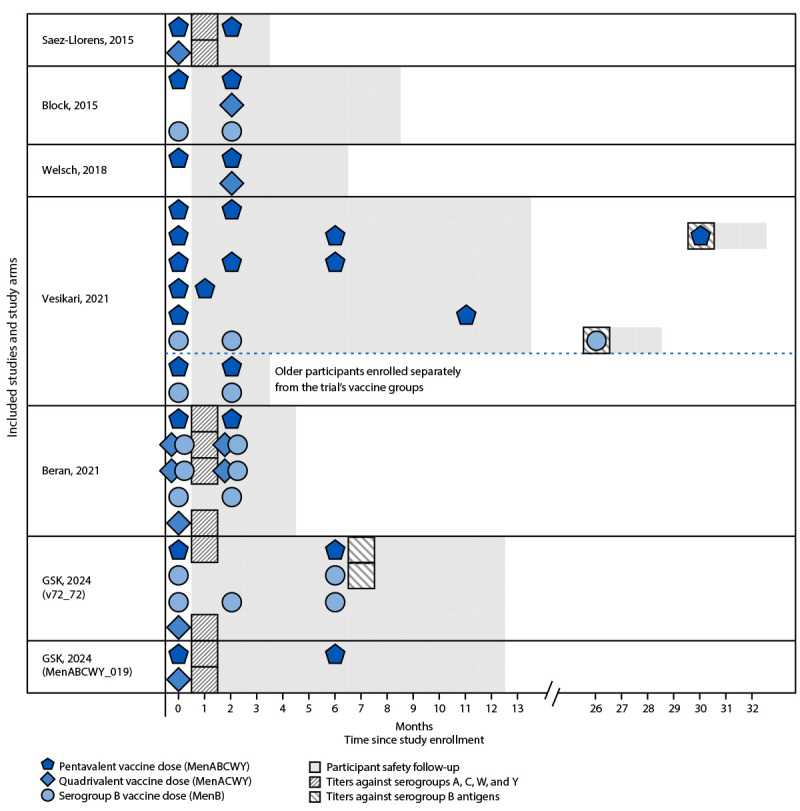
Summary of vaccine studies,* vaccine groups,^†^ immunogenicity timepoints,^§^ and safety follow-up^¶^ included in the assessment of evidence for use of pentavalent (MenABCWY) vaccine — United States, 2025 * Saez-Llorens, 2015: https://doi.org/10.1080/21645515.2015.1029686; Block, 2015: https://doi.org/10.1016/j.vaccine.2015.03.001; Welsch, 2018: https://doi.org/10.1016/j.vaccine.2018.07.016; Vesikari, 2021: https://doi.org/10.1080/21645515.2021.1968214; Beran, 2021: https://doi.org/10.1128/mSphere.00553-21; GSK, 2024 (v72_72): https://clinicaltrials.gov/study/NCT04502693; GSK, 2024 (MenABCWY_019): https://clinicaltrials.gov/study/NCT04707391. For the Beran (2021) study, the two vaccine groups with concomitant administration of MenACWY and MenB doses differed slightly; participants of one group received both doses in the same arm, whereas the other received each dose in a different arm. ^†^ Only vaccine groups considered in the evidence assessment are depicted. When multiple doses were administered, the per-protocol interval between doses is depicted. ^§^ Immunogenicity timepoints are the timepoints considered in the evidence assessment, not all protocol-specified timepoints at which immunogenicity was evaluated. ^¶^ Safety follow-up refers to the follow-up period for protocol-specified reporting of serious adverse events.

### Short-Term (≤1 Year Postvaccination) Immunity

Four studies contributed evidence for short-term immunogenicity against serogroups A, C, W, and Y. In one, participants in the MenACWY-CRM control group received doses of MenACWY-CRM and MenB-4C at the same study visit, while control group participants in the other three studies had only received MenACWY-CRM before titers were assessed. Two studies required individuals to have no prior history of MenACWY receipt, one study required individuals to have previously received MenACWY, and one did not have a requirement regarding MenACWY history. One month after receipt of 1 dose of ACWY–targeting vaccine, participants in the pentavalent group (MenACWY-CRM/MenB-4C) and control group (MenACWY-CRM) were similarly likely* to have seroprotective titers against serogroups A, C, W, and Y.

One month after receipt of 2 doses of serogroup B–targeting vaccine administered 6 months apart, participants in the pentavalent group (MenACWY-CRM/MenB-4C) and control group (only MenB-4C, with no concomitant administration of MenACWY-CRM) were similarly likely^†^ to have seroprotective antibody titers against three of four serogroup B antigens (fHbp, NadA, and NHBA). Participants in the pentavalent group were less likely than control group participants were to have seroprotective antibody titers against the fourth antigen, PorA. PorA is thought to be responsible for broad cross-protection against various serogroup B strains; however, the clinical implications of the reduced PorA response are unknown, as differences in titer assay choices and circulating strains make comparing data across studies complex (*14*,*15*). In addition, whether the reduced response might result from immune interference could not be assessed because of limited data comparing titers after 2 doses given 2 months apart, rather than 6 months apart. The overall level of certainty for the evidence regarding short-term immunity was moderate for healthy persons and low for persons at increased risk for meningococcal disease (*5*).

### Persistent (>1 Year Postvaccination) Immunity 

No identified studies evaluated persistence of immunity against serogroups A, C, W, and Y. Two years after receipt of 2 doses of serogroup B–targeting vaccine, participants in the pentavalent group (MenACWY-CRM/MenB-4C doses administered 6 months apart) and the control group (MenB-4C doses administered 2 months apart) were similarly likely to have seroprotective antibody titers against all four serogroup B antigens. The overall level of certainty for the evidence regarding persistent immunity was low for healthy persons and very low for persons at increased risk for meningococcal disease (*5*).

### Adverse Events

A serious adverse event (SAE) is an untoward medical occurrence that results in death, disability, or incapacity; is life threatening; or requires hospitalization or prolongation of existing hospitalization. The possible relationship between an event and the study vaccine was judged by the study investigator, not the study sponsor. Certain studies also included an untoward medical occurrence that resulted in a congenital anomaly or birth defect in the offspring of a participant in SAE assessments. Solicited adverse events (e.g., injection site redness or pain, fever, or fatigue) were used as a surrogate for nonserious adverse events.

SAEs possibly related to vaccination after any vaccine dose were rare overall and were similarly frequent among participants in the pentavalent (MenACWY-CRM/MenB-4C) and control (MenACWY-CRM, MenB-4C, or both) groups. Six SAEs possibly related to vaccination occurred among 7,847 participants enrolled in relevant vaccine groups considered in the evidence assessment across all seven studies, including three in the pentavalent group (seizure, connective tissue disorder, and neuromyelitis optica among 3,925 participants) and three in the control groups (syncope [MenB-4C group], pyrexia [MenACWY-CRM group], and ulcerative colitis [MenB-4C group] among 3,922 participants). The events of neuromyelitis optica, pyrexia, and ulcerative colitis were assessed as related to study vaccination by the study investigators; however, after evaluations by GSK and an independent evaluator, this condition was not considered an adverse drug reaction. The overall level of certainty for the evidence regarding frequency of SAEs possibly related to vaccination was moderate for healthy persons and low for persons at increased risk for meningococcal disease (*5*).

Persons in the pentavalent group (MenACWY-CRM/MenB-4C) and serogroup B–targeting control groups (MenB-4C or MenACWY-CRM + MenB-4C) were similarly likely^§^ to experience or report one or more nonserious adverse events after 1 or ≥2 doses. The overall level of certainty for the evidence regarding frequency of nonserious adverse events after 1 dose of pentavalent versus MenB alone or ≥2 doses of pentavalent versus MenB alone was high for healthy persons and moderate for persons at increased risk for meningococcal disease (*5*). The overall level of certainty for the evidence regarding frequency of nonserious adverse events after 1 dose of pentavalent versus 1 dose of MenB concomitantly with 1 dose of MenACWY was moderate for healthy persons and low for persons at increased risk for meningococcal disease (*5*).

Compared with persons who received a single MenACWY-CRM dose, those in the pentavalent group were significantly more likely^¶^ to experience or report nonserious adverse events after either 1 dose or 2 doses administered 6 months apart. The overall level of certainty for the evidence regarding frequency of nonserious adverse events after 1 dose of pentavalent versus 1 dose of MenACWY and ≥2 doses of pentavalent versus 1 dose of MenACWY was moderate for healthy persons and low for persons at increased risk for meningococcal disease (*5*). 

### Resource Use

To assess the cost-effectiveness of MenACWY-CRM/MenB-4C for each policy question, ACIP considered findings from a CDC model (*16*,*17*) and a GSK model (*18*). The CDC model estimated that pentavalent vaccines would be cost-saving under policy question 1 (replacing MenACWY and MenB when both are indicated). Under policy question 2 (replacing MenACWY), pentavalent vaccine would cost $11.3 million per QALY gained. For policy question 3 (replacing MenB), the cost-effectiveness of pentavalent vaccine would range from being cost-saving (though less cost-saving than policy question 1) to costing $4.5 million per QALY (*16*,*17*). Using pentavalent vaccine as an alternative to concomitant administration of MenACWY and MenB (policy question 1) was the most cost-saving of the policy questions under consideration. The GSK model included several differences in assumptions and inputs but yielded similar conclusions overall (*18*).

## Recommendations for Use of MenACWY-CRM/MenB-4C

ACIP recommended that MenACWY-CRM/MenB-4C may be used when both MenACWY and MenB are indicated at the same visit for 1) healthy persons aged 16–23 years (routine schedule) when shared clinical decision-making favors administration of MenB vaccine and 2) persons aged ≥10 years who are at increased risk for meningococcal disease (e.g., because of persistent complement deficiencies, complement inhibitor use, or functional or anatomic asplenia). Indications for MenACWY and MenB vaccination have not changed since they were published in 2020 (*2*).

### Clinical Guidance

**Shared clinical decision-making for MenB.** For healthy persons, MenACWY-CRM/MenB-4C may be administered on the basis of shared clinical decision-making. Providers can refer to previously published considerations for shared clinical decision-making regarding MenB administration (*3*).

**Interchangeability of vaccine products.** MenACWY products are interchangeable, although the same vaccine product is preferred for all doses (*2*). However, because MenB products from different manufacturers contain different antigens, they are not interchangeable; existing recommendations for administering MenB products are based on evidence for which only the same manufacturer’s product was administered. All doses of a serogroup B–targeting vaccine administered to an individual person, including booster doses, should be from the same manufacturer. If doses from multiple manufacturers have been administered to the same person, the person should receive a complete series of either manufacturer’s product without counting doses from the other manufacturer as valid (*6*). Providers should always check the packaging label to be certain they are administering the intended vaccine product to the recipient (*19*).

If MenB doses have been previously received by an individual but the vaccine manufacturer is unknown, an attempt should be made to obtain past immunization records (e.g., from the health care provider or immunization information system [immunization registry]) to ascertain the vaccine brand used for previous MenB doses. If the brand cannot be determined, the series should be restarted with any licensed MenB–targeting vaccine to ensure receipt of a complete MenB series with products from a single manufacturer.

**Indavertent administration of MenACWY-CRM/MenB-4C.** If MenACWY-CRM/MenB-4C is inadvertently administered when either MenACWY only or MenB only is indicated, the dose can be considered valid if it otherwise would have been a valid administration of MenACWY or MenB.

**Dosing intervals.** Healthy adolescents and young adults aged 16–23 years who receive a dose of MenACWY-CRM/MenB-4C on the basis of shared clinical decision-making should complete the MenB series with a dose of MenB-4C administered 6 months after the MenACWY-CRM/MenB-4C dose (*3*). Persons at increased risk for meningococcal disease who receive a dose of MenACWY-CRM/MenB-4C and who are recommended to receive additional doses of MenACWY and MenB <6 months after a dose of pentavalent meningococcal vaccine should receive separate MenACWY and MenB-4C vaccines, rather than MenACWY-CRM/MenB-4C, following recommended intervals (*3*). Additional details regarding when doses might need to be administered <6 months apart are available in the Child Immunization Schedule Notes (Meningococcal Serogroup A, C, W, Y Vaccination and Meningococcal Serogroup B Vaccination) and the Adult Immunization Schedule Notes (Meningococcal Vaccination).

MenACWY-CRM/MenB-4C may be used for booster doses among persons at increased risk for meningococcal disease if a booster dose of both MenACWY and MenB are indicated at the same visit. MenACWY-CRM/MenB-4C doses deviating from the licensed 6-month interval can be considered valid for MenACWY or MenB if the timing would otherwise have been valid for that component.

### Contraindications and Precautions

**Severe allergy.** MenACWY-CRM/MenB-4C is contraindicated for persons who have a history of a severe allergic reaction, such as anaphylaxis, to any component of the vaccine or to a diphtheria toxoid–containing vaccine (Penmenvy [Meningococcal Groups A, B, C, W, and Y vaccine] | Highlights of Prescribing Information).

**Pregnancy and breastfeeding.** No high-quality data have been published on use of MenACWY-CRM/MenB-4C during pregnancy or while breastfeeding. Because data regarding MenB vaccination during pregnancy are limited, vaccination with MenB during pregnancy should be deferred until after pregnancy unless an increased risk for acquiring meningococcal disease exists and, after consulting a health care provider, the benefits of vaccination are considered to outweigh the potential risks. When MenACWY is indicated while pregnant or breastfeeding, MenACWY-CRM or MenACWY-TT may be administered.

**Moderate or severe acute illness.** As with other vaccines, vaccination should generally be deferred for persons with a moderate or severe acute illness.

### Reporting of Vaccine Adverse Events

Adverse events that occur in a patient after meningococcal vaccination should be reported to the Vaccine Adverse Event Reporting System (VAERS), even if it is uncertain whether the vaccine caused the event. Instructions for reporting to VAERS are available online at VAERS | Report an Adverse Event or by telephone (800-822-7967).

## Footnotes

* This judgment is based on the 95% CIs for the relative effect on seroprotective titers when comparing MenABCWY with MenACWY: 0.86–1.01 for serogroup A, 0.97–1.10 for serogroup C, 1.00–1.04 for serogroup W, and 0.93–1.03 for serogroup Y. GSK’s Pentavalent Meningococcal Vaccine | MenACWY-CRM/MenB-4C | GRADE

† This judgment is based on the 95% CIs for the relative effect on seroprotective titers when comparing MenABCWY with MenB: 0.99–1.04 for fHbp, 0.96–1.00 for both NadA and NHBA, and 0.86–0.96 for PorA. GSK’s Pentavalent Meningococcal Vaccine | MenACWY-CRM/MenB-4C | GRADE

§ This judgment is based on the 95% CIs for the relative risk of experiencing one or more nonserious adverse event when comparing MenABCWY with serogroup B–targeting control groups: 0.96–1.02 for 1 dose of MenABCWY versus 1 dose of MenB only, 0.90–1.01 for 1 dose of MenABCWY versus 1 dose of MenACWY + MenB, and 0.98–1.02 for ≥2 doses of MenABCWY versus ≥2 doses of MenB.

¶ This judgment is based on the 95% CIs for the relative risk of experiencing one or more nonserious adverse event when comparing MenABCWY with MenACWY: 1.46–2.22 for 1 dose of MenABCWY versus 1 dose of MenACWY and 1.83–2.21 for ≥2 doses of MenABCWY versus 1 dose of MenACWY.
